# Luxation de l’épaule compliquée de paralysie du plexus brachial

**DOI:** 10.11604/pamj.2014.18.229.4869

**Published:** 2014-07-18

**Authors:** Loubet Unyendje Lukulunga, Abdou Kadri Moussa, Mustapha Mahfoud, Ahmed EL Bardouni, Mohamed Saleh Berrada, Moradh El Yaacoubi

**Affiliations:** 1 Service de Traumatologie Orthopédie, Centre Hospitalier Universitaire Ibn Sina, Rabat, Maroc

**Keywords:** Luxation, épaule, paralysie, plexus brachial, dislocation, shoulder, palsy, brachial plexus

## Abstract

Les auteurs rapportent l'observation d'une paralysie totale du plexus brachial survenue trois mois après un épisode de luxation antéro-interne sous coracoïdienne associée à une fracture du trochiter chez une patiente âgée de 88 ans.

## Introduction

La survenue des paralysies totales du plexus brachial post luxation de l’épaule est un désastre pour le patient, la famille et la société dont le pronostic fonctionnel du membre ou le retour à un état antérieur n'est pas un objectif réaliste. C'est un handicap majeur par perte d'autonomie, du travail et du sport et aussi une charge sociale nécessitant une aide permanente. Les luxations de l’épaule sont devenues de plus en plus fréquentes soit 27/100 000 habitants suite aux accidents de voie publique et au développement des activités sportives. Elles peuvent être pures ou associées à une fracture de la tête humérale ou de la cavité glénoïde et peuvent se compliquer d'atteinte vasculaire ou neurologique. 21 à 62% des atteintes neurologiques surviennent après luxations chez les jeunes [1, 2]. Cette association lésionnelle a été décrite pour la première fois en 1910 par Delbet et Couchoix [3]. Sa prise en charge reste un grand défi en traumatologie car le patient n'attend qu'une chose la récupération complète de son membre; l'avènement de nouvelles techniques d'exploration dont l’électromyographie(EMG), l'imagerie par résonance magnétique(IRM) et l'introduction de la microchirurgie ont révolutionné la prise dans le monde [4]. Le but de ce travail est de discuter sur les particularités de cette triple association lésionnelle et de faire une revue de la littérature.

## Patient et observation

**Figure 1:**
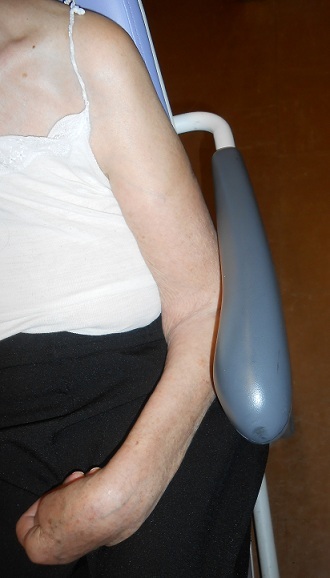
Aspects cliniques d'atteinte sévère du plexus brachial montrant un défaut d'extension du poignet et des doigts avec une impotence fonctionnelle totale du membre supérieur gauche

**Figure 2:**
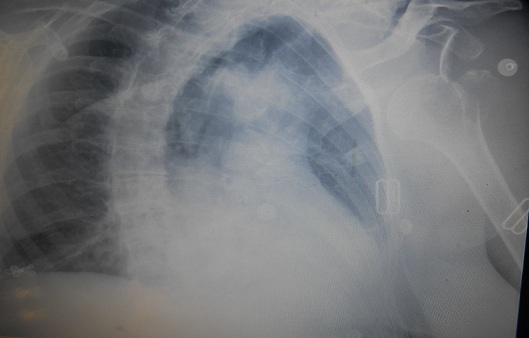
La radiographie de l’épaule objectivant une luxation antéro-interne sous coracoïdienne avec arrachement de trochiter

**Figure 3:**
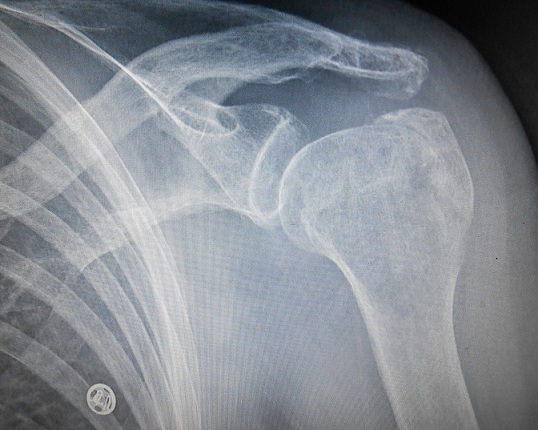
La radiographie de face à 3 mois de la réduction

Il s'agit d'une patiente âgée de 88 ans admise en consultation d'orthopédie de l'hôpital pour trouble de sensibilité, d'extension du poignet et des doigts avec une impotence fonctionnelle totale du membre supérieur gauche(Figure 1). L'histoire remonte à trois mois par une chute sur l’épaule gauche dont la radiographie de face et de profil a révélé une luxation antéro-interne sous coracoïdienne avec arrachement de trochiter (Figure 2). La réduction était faite en urgence selon la technique d'Astley-Cooper qui a consisté au bloc opératoire sous anesthésie générale à porter tout doucement le bras en élévation, puis en abduction et en rotation externe pendant que l'autre main pousse sur la tête qui est dans l'aisselle (Figure 3). Suivi d'une immobilisation coude au corps pendant 6 semaines associée à la rééducation. Malgré la kinésithérapie la patiente se plaignait des fourmillements et des picotements le long du membre depuis sa chute. Dans ses antécédents on a noté une notion d'AVC sans séquelle survenu il y a 12 ans et d'une fracture du col fémoral opérée il y a 2 ans. L'examen clinique a trouvé une patiente consciente en bon état général en attitude vicieuse du membre supérieur gauche avec coude, poignet et doigts en flexion (Figure 1) dont toute tentative d'extension paraissait difficile; On a noté un appendement et une amyotrophie des muscles de l’épaule avec un œdème prenant le godet au niveau de la face dorsale de la main gauche. La palpation du membre reveille une douleur à type de décharge électrique. Les amplitudes articulaires de la gléno-humérale sont limitées et les doigts sont complètement fléchis. L'examen vasculaire était sans particularité. Une IRM cérébrale pour suspicion d'AVC était sans particularité, L'EMG a confirmé une atteinte sévère du plexus brachial. Le traitement a consisté à une rééducation au long cours et la récupération fonctionnelle n’était que partielle.

## Discussion

Les luxations de l’épaule peuvent s'associer à une paralysie totale du plexus brachial ou à une fracture du trochiter. Cette association lésionnelle a été décrite pour la première fois en 1910 par Delbet et Couchoix.

Elles surviennent souvent lors des accidents de voie publique par un traumatisme violent de la ceinture scapulaire chez les jeunes motocyclistes. Alieu et al. dans leur série avaient trouvé 90% de paralysies brachiales post traumatiques chez les jeunes motocyclistes [5]. Pour notre cas l’âge de notre patiente était de 88 ans et qui suite à une chute mécanique de sa hauteur sur la main en abduction et en rotation interne a présenté une fracture-luxation de l’épaule. Quelque soit l’âge, et la cinétique de la force traumatisante, le passage du plexus brachial en avant, en dedans, et en bas par rapport à l'articulation gléno-humérale fait qu'en cas de déplacement de la tête humérale en dehors de la cavité glénoïde pourrait entrainer soit une compression ou un étirement avec arrachement des racines du plexus brachial. En plus les hémorragies survenues lors de la luxation et de la fracture du trochiter pourraient constituer un hématome responsable de la compression du plexus brachial et de l'hyperpression aigüe du canal avec gène de la micro-circulation veineuse qui va être à l'origine d'un œdème intra neural. Cet œdème va s'organiser plus tard en fibrose et en exsudat qui engainent les fibres nerveuses. Ces exsudats vont diffuser à travers les vaisseaux épi neuraux et entrainer l'ischémie qui va altérer la conduction nerveuse [6, 7]. C'est ainsi qu'en rétablissant la circulation sanguine du nerf en urgence, la fonction nerveuse se rétablit aussi dans un délai qui coïncide avec la revascularisation endoneural. Chez les sujets âgés l'artériosclérose des vaisseaux au niveau épi neuraux pourraient accentuer l'ischémie qui à son tour va altérer la conduction nerveuse. La prise en charge des paralysies brachiales post-traumatiques reste un grand défi en traumatologie par l'absence de consensus dans le traitement chirurgical surtout chez les sujets âgés. Les uns proposent une exploration chirurgicale avec neurolyse en l'absence de récupération après 6 à 12 mois [8]. Les autres en cas de rupture préfèrent une greffe nerveuse [9], et les derniers souhaitent un transfert tendineux de réanimation de la musculature intrinsèque de la main [10]. L'attitude moderne face aux avulsions et aux neurotmesis reste un diagnostic précoce, rapide et une reconstruction micro-chirurgicale [11]. Pour notre patiente la chirurgie à envisager ne pouvait être que palliative cependant compte tenu de la fibrose cicatricielle elle n'a pu bénéficier que de la kinésithérapie.

## Conclusion

La prise en charge de paralysies brachiales post- traumatiques chez les sujets âgés reste un grand défi en traumatologie par l'absence de consensus dans le traitement chirurgical. L'attitude actuelle reste un diagnostic précoce et rapide avec l'avènement de nouvelles techniques d'exploration dont l’électromyographie (EMG), l'imagerie par résonance magnétique (IRM) ainsi que la reconstruction microchirurgicale qui pourraient améliorer le pronostic du patient qui n'attend qu'une chose la récupération complète de son membre.
